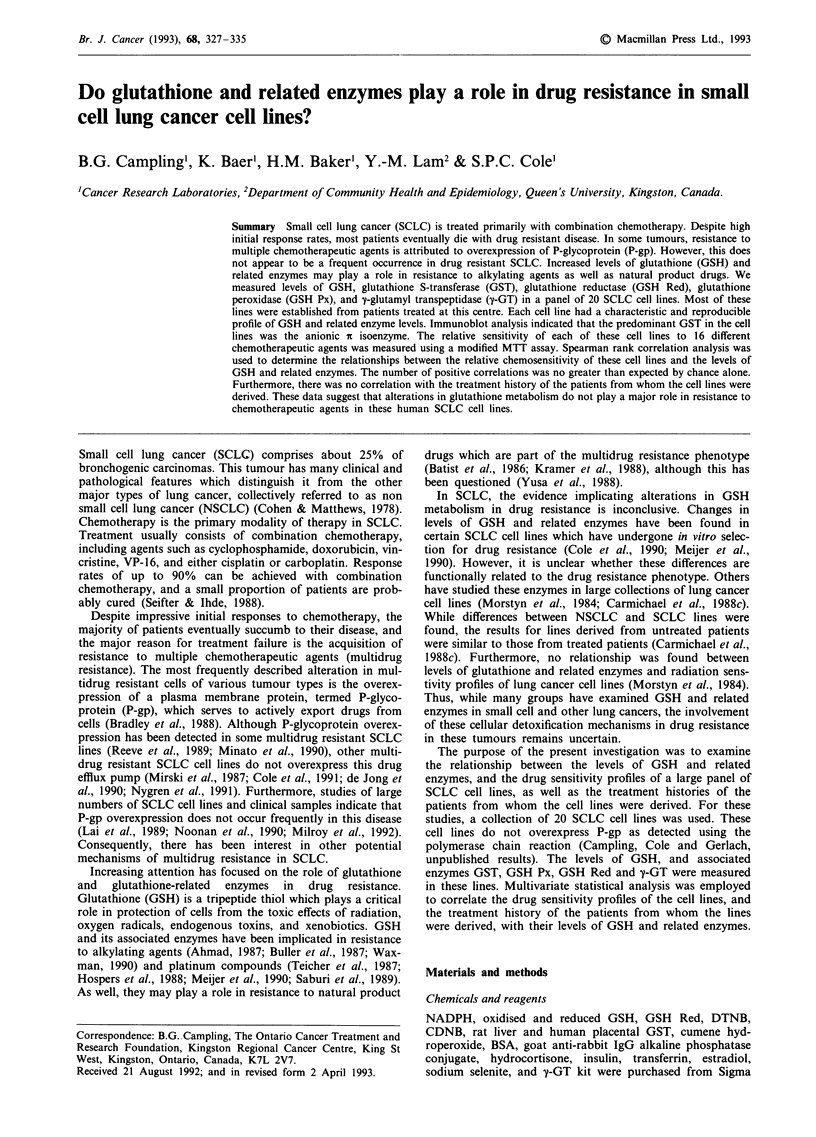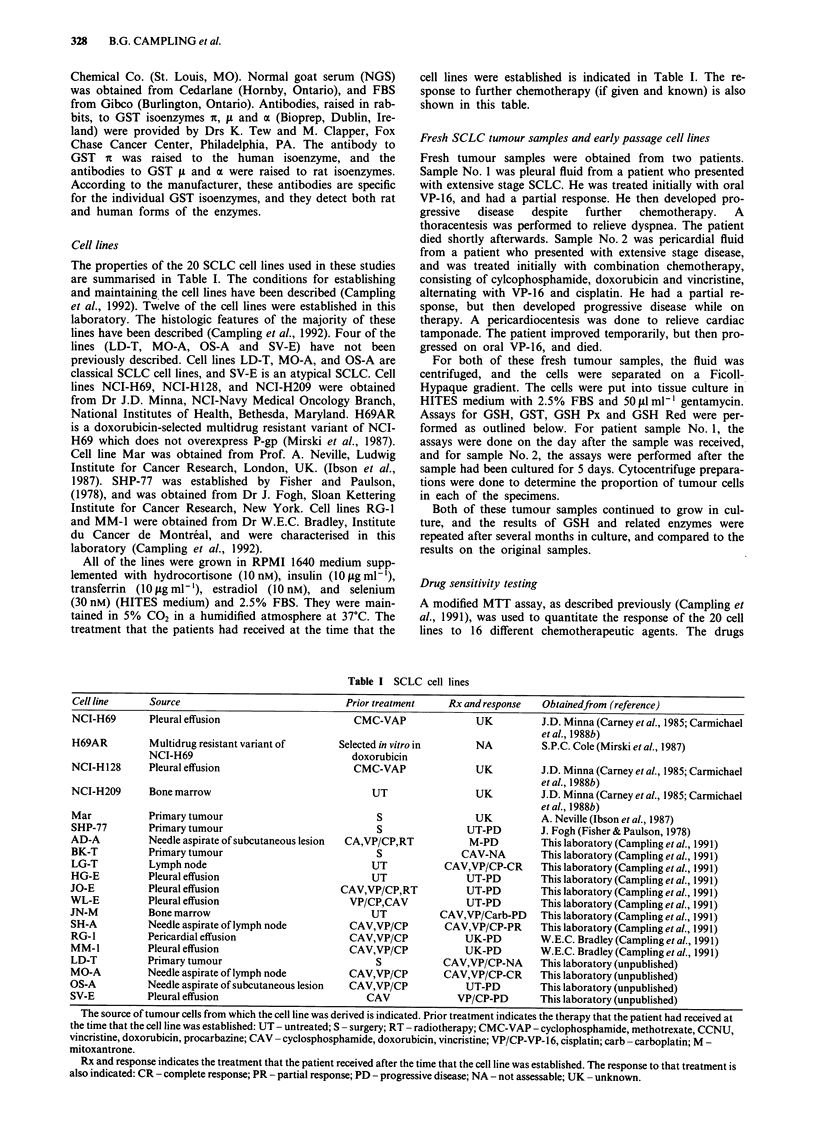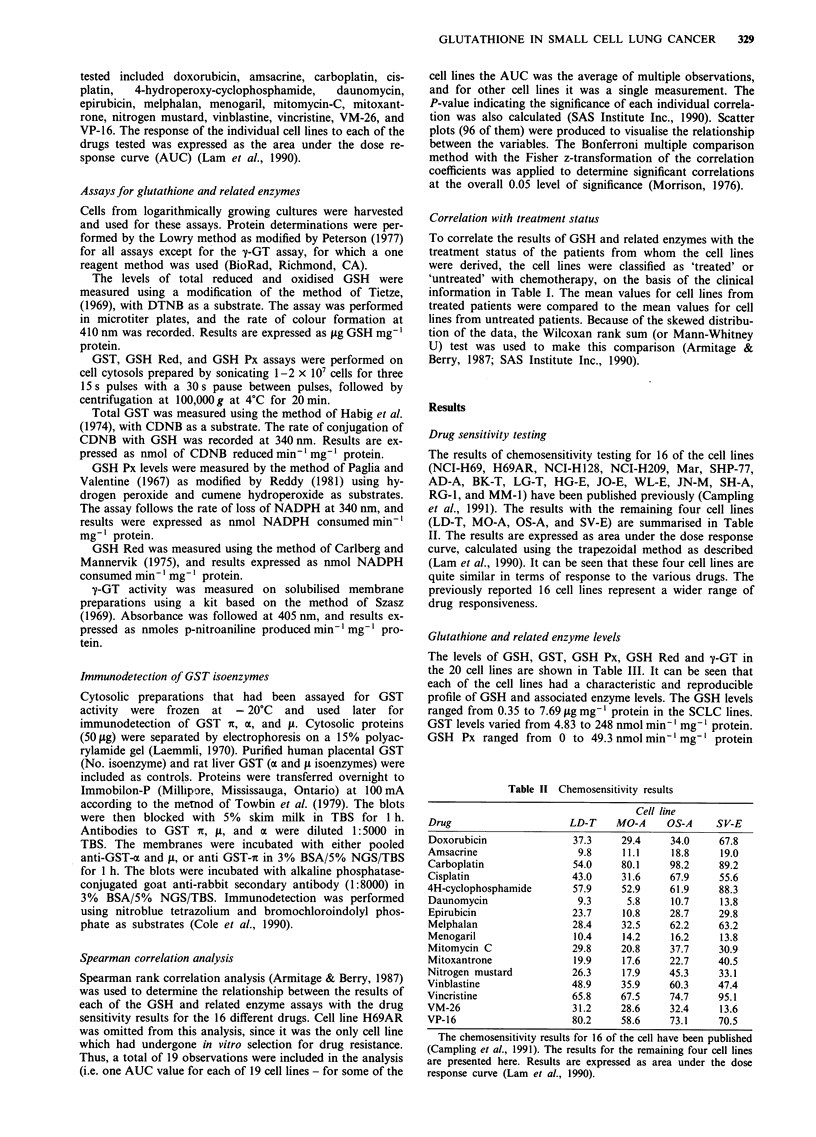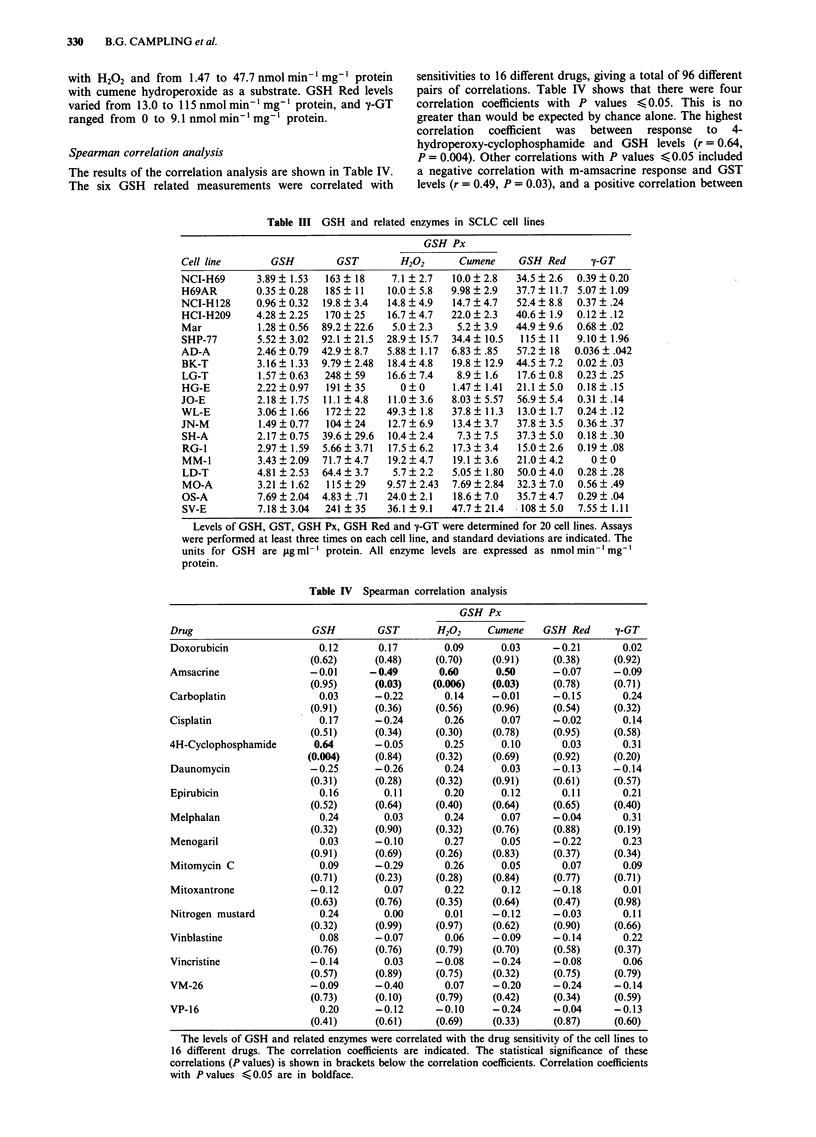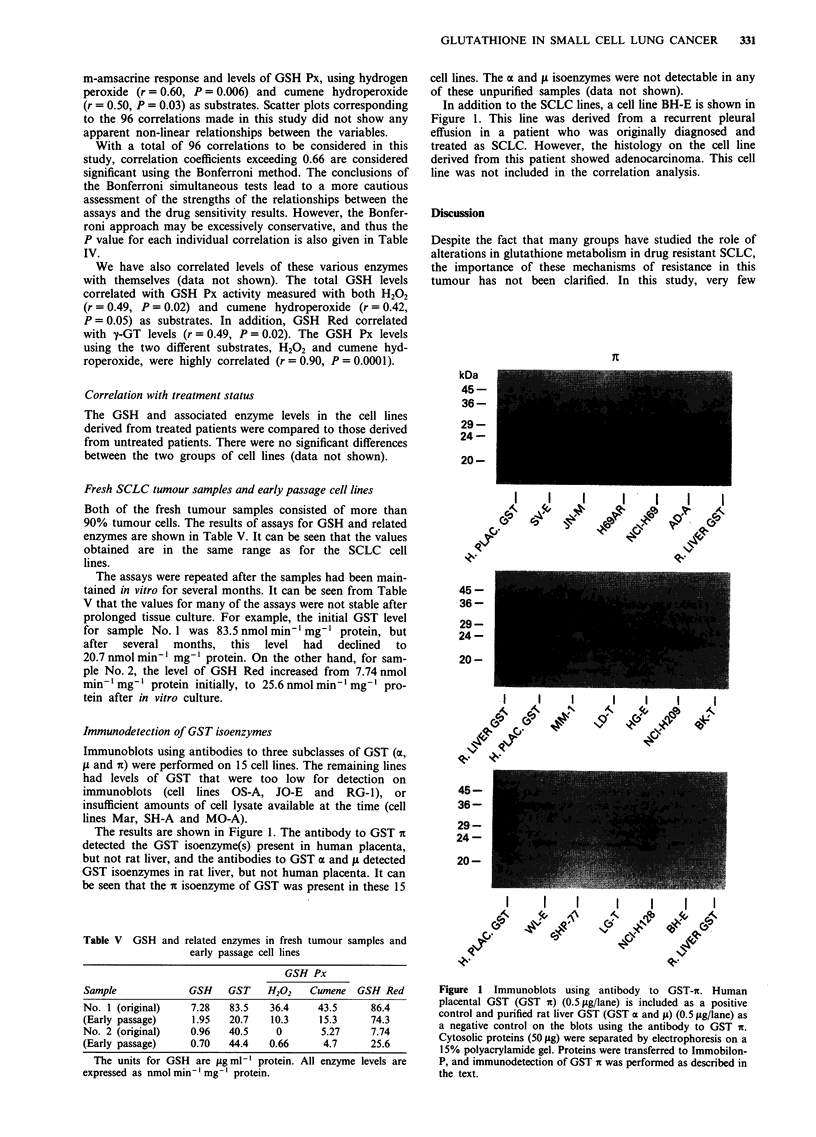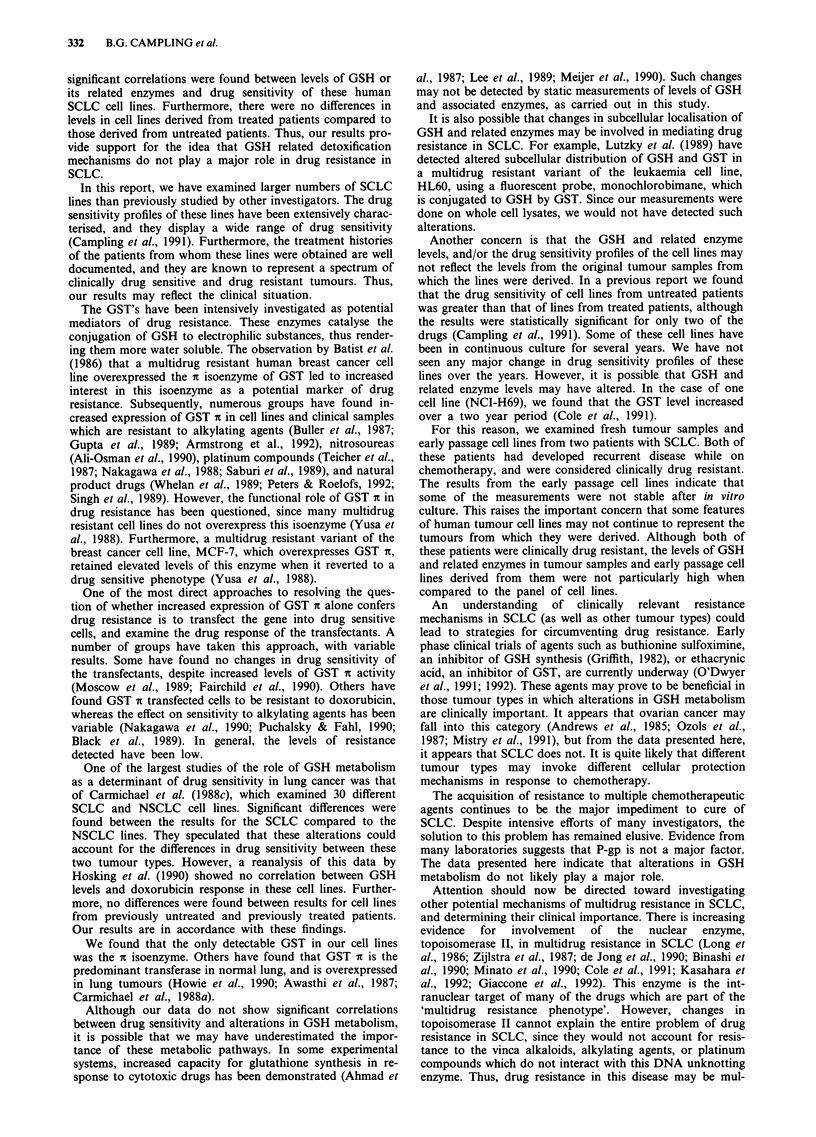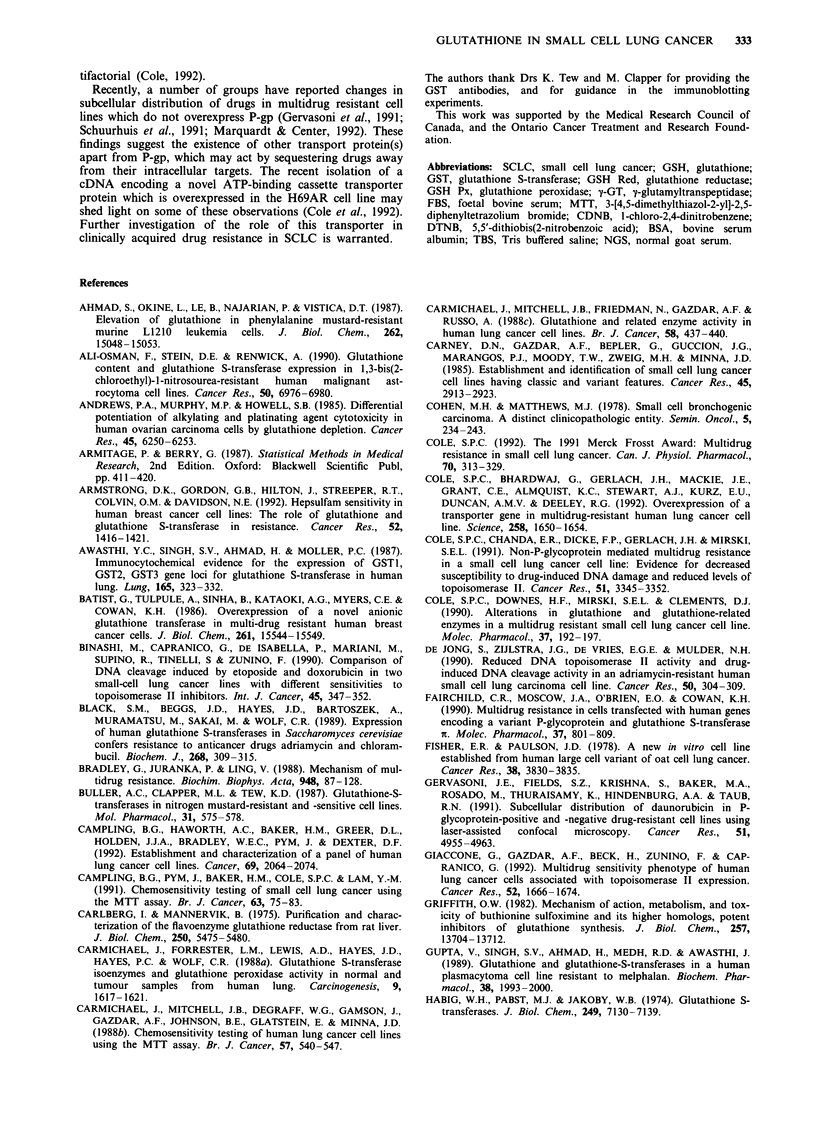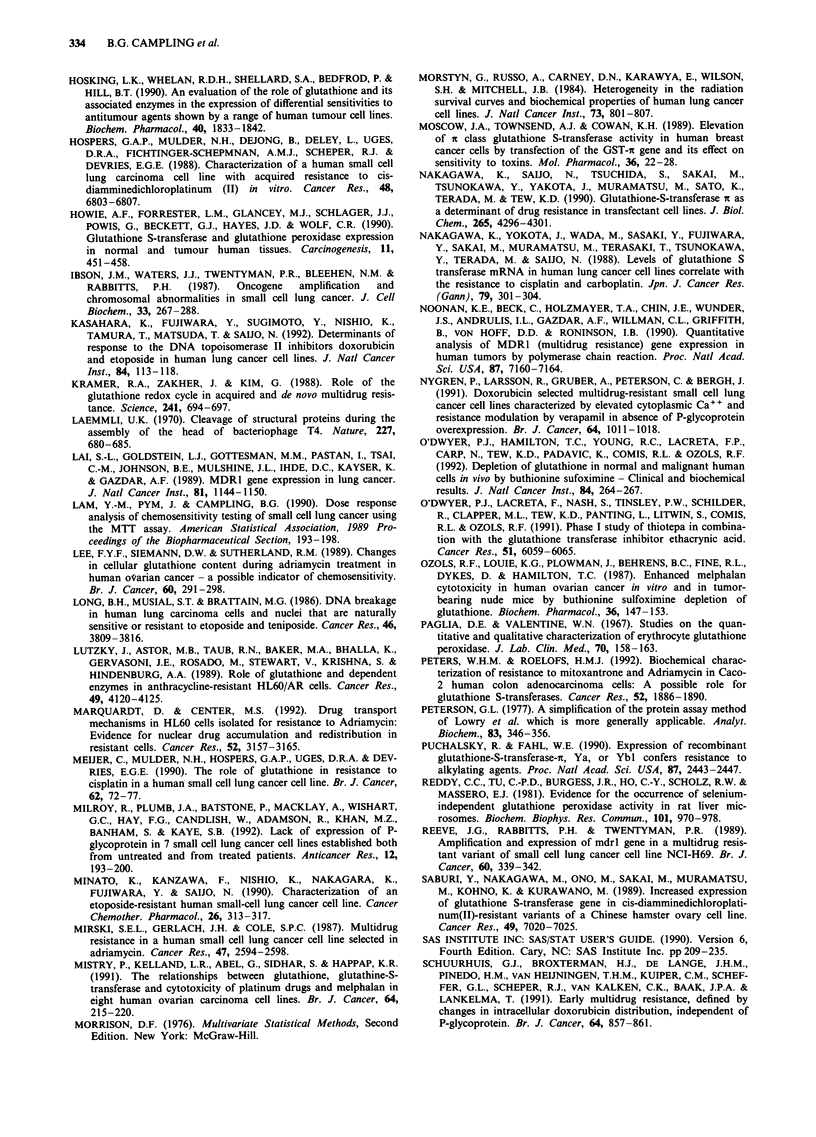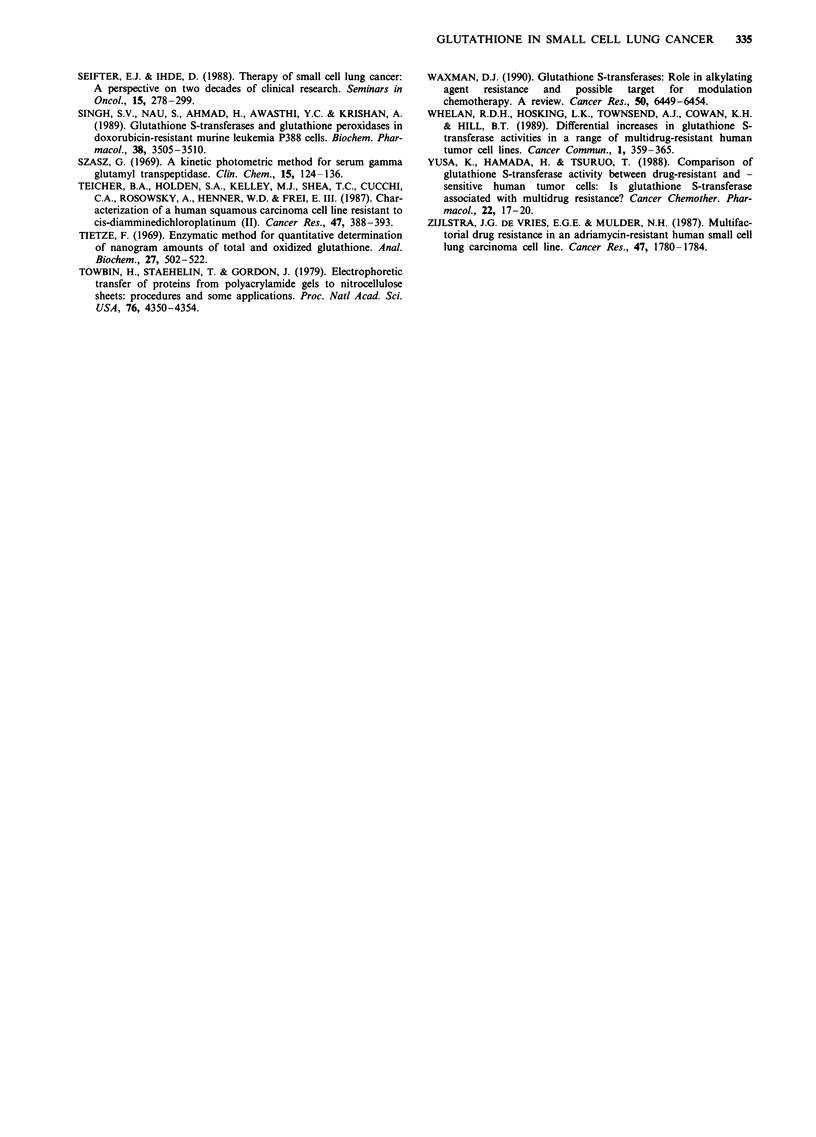# Do glutathione and related enzymes play a role in drug resistance in small cell lung cancer cell lines?

**DOI:** 10.1038/bjc.1993.336

**Published:** 1993-08

**Authors:** B. G. Campling, K. Baer, H. M. Baker, Y. M. Lam, S. P. Cole

**Affiliations:** Cancer Research Laboratories, Queen's University, Kingston, Canada.

## Abstract

**Images:**


Br. J. Cancer (1993), 68, 327–335 © Macmillan Press Ltd., 1993

### Do glutathione and related enzymes play a role in drug resistance in small cell lung cancer cell lines?

B.G. Campling[1], K. Baer[1], H.M. Baker[1], Y.-M. Lam[2] & S.P.C. Cole[1]

[1]*Cancer Research Laboratories,* [2]*Department of Community Health and Epidemiology, Queen's University, Kingston, Canada.*

**Summary** Small cell lung cancer (SCLC) is treated primarily with combination chemotherapy. Despite high initial response rates, most patients eventually die with drug resistant disease. In some tumours, resistance to multiple chemotherapeutic agents is attributed to overexpression of P-glycoprotein (P-gp). However, this does not appear to be a frequent occurrence in drug resistant SCLC. Increased levels of glutathione (GSH) and related enzymes may play a role in resistance to alkylating agents as well as natural product drugs. We measured levels of GSH, glutathione S-transferase (GST), glutathione reductase (GSH Red), glutathione peroxidase (GSH Px), and γ-glutamyl transpeptidase (γ-GT) in a panel of 20 SCLC cell lines. Most of these lines were established from patients treated at this centre. Each cell line had a characteristic and reproducible profile of GSH and related enzyme levels. Immunoblot analysis indicated that the predominant GST in the cell lines was the anionic π isoenzyme. The relative sensitivity of each of these cell lines to 16 different chemotherapeutic agents was measured using a modified MTT assay. Spearman rank correlation analysis was used to determine the relationships between the relative chemosensitivity of these cell lines and the levels of GSH and related enzymes. The number of positive correlations was no greater than expected by chance alone. Furthermore, there was no correlation with the treatment history of the patients from whom the cell lines were derived. These data suggest that alterations in glutathione metabolism do not play a major role in resistance to chemotherapeutic agents in these human SCLC cell lines.

Small cell lung cancer (SCLC) comprises about 25% of bronchogenic carcinomas. This tumour has many clinical and pathological features which distinguish it from the other major types of lung cancer, collectively referred to as non small cell lung cancer (NSCLC) (Cohen & Matthews, 1978). Chemotherapy is the primary modality of therapy in SCLC. Treatment usually consists of combination chemotherapy, including agents such as cyclophosphamide, doxorubicin, vincristine, VP-16, and either cisplatin or carboplatin. Response rates of up to 90% can be achieved with combination chemotherapy, and a small proportion of patients are probably cured (Seifter & Ihde, 1988).

Despite impressive initial responses to chemotherapy, the majority of patients eventually succumb to their disease, and the major reason for treatment failure is the acquisition of resistance to multiple chemotherapeutic agents (multidrug resistance). The most frequently described alteration in multidrug resistant cells of various tumour types is the overexpression of a plasma membrane protein, termed P-glycoprotein (P-gp), which serves to actively export drugs from cells (Bradley *et al.*, 1988). Although P-glycoprotein overexpression has been detected in some multidrug resistant SCLC lines (Reeve *et al.*, 1989; Minato *et al.*, 1990), other multidrug resistant SCLC cell lines do not overexpress this drug efflux pump (Mirski *et al.*, 1987; Cole *et al.*, 1991; de Jong *et al.*, 1990; Nygren *et al.*, 1991). Furthermore, studies of large numbers of SCLC cell lines and clinical samples indicate that P-gp overexpression does not occur frequently in this disease (Lai *et al.*, 1989; Noonan *et al.*, 1990; Milroy *et al.*, 1992). Consequently, there has been interest in other potential mechanisms of multidrug resistance in SCLC.

Increasing attention has focused on the role of glutathione and glutathione-related enzymes in drug resistance. Glutathione (GSH) is a tripeptide thiol which plays a critical role in protection of cells from the toxic effects of radiation, oxygen radicals, endogenous toxins, and xenobiotics. GSH and its associated enzymes have been implicated in resistance to alkylating agents (Ahmad, 1987; Buller *et al.*, 1987; Waxman, 1990) and platinum compounds (Teicher *et al.*, 1987; Hospers *et al.*, 1988; Meijer *et al.*, 1990; Saburi *et al.*, 1989). As well, they may play a role in resistance to natural product drugs which are part of the multidrug resistance phenotype (Batist *et al.*, 1986; Kramer *et al.*, 1988), although this has been questioned (Yusa *et al.*, 1988).

In SCLC, the evidence implicating alterations in GSH metabolism in drug resistance is inconclusive. Changes in levels of GSH and related enzymes have been found in certain SCLC cell lines which have undergone *in vitro* selection for drug resistance (Cole *et al.*, 1990; Meijer *et al.*, 1990). However, it is unclear whether these differences are functionally related to the drug resistance phenotype. Others have studied these enzymes in large collections of lung cancer cell lines (Morstyn *et al.*, 1984; Carmichael *et al.*, 1988*c*). While differences between NSCLC and SCLC lines were found, the results for lines derived from untreated patients were similar to those from treated patients (Carmichael *et al.*, 1988*c*). Furthermore, no relationship was found between levels of glutathione and related enzymes and radiation senstivity profiles of lung cancer cell lines (Morstyn *et al.*, 1984). Thus, while many groups have examined GSH and related enzymes in small cell and other lung cancers, the involvement of these cellular detoxification mechanisms in drug resistance in these tumours remains uncertain.

The purpose of the present investigation was to examine the relationship between the levels of GSH and related enzymes, and the drug sensitivity profiles of a large panel of SCLC cell lines, as well as the treatment histories of the patients from whom the cell lines were derived. For these studies, a collection of 20 SCLC cell lines was used. These cell lines do not overexpress P-gp as detected using the polymerase chain reaction (Campling, Cole and Gerlach, unpublished results). The levels of GSH, and associated enzymes GST, GSH Px, GSH Red and γ-GT were measured in these lines. Multivariate statistical analysis was employed to correlate the drug sensitivity profiles of the cell lines, and the treatment history of the patients from whom the lines were derived, with their levels of GSH and related enzymes.

### Materials and methods

### *Chemicals and reagents*

NADPH, oxidised and reduced GSH, GSH Red, DTNB, CDNB, rat liver and human placental GST, cumene hydroperoxide, BSA, goat anti-rabbit IgG alkaline phosphatase conjugate, hydrocortisone, insulin, transferrin, estradiol, sodium selenite, and γ-GT kit were purchased from Sigma

Correspondence: B.G. Campling, The Ontario Cancer Treatment and Research Foundation, Kingston Regional Cancer Centre, King St West, Kingston, Ontario, Canada, K7L 2V7.
Received 21 August 1992; and in revised form 2 April 1993.

Chemical Co. (St. Louis, MO). Normal goat serum (NGS) was obtained from Cedarlane (Hornby, Ontario), and FBS from Gibco (Burlington, Ontario). Antibodies, raised in rabbits, to GST isoenzymes π, μ and α (Bioprep, Dublin, Ireland) were provided by Drs K. Tew and M. Clapper, Fox Chase Cancer Center, Philadelphia, PA. The antibody to GST π was raised to the human isoenzyme, and the antibodies to GST μ and α were raised to rat isoenzymes. According to the manufacturer, these antibodies are specific for the individual GST isoenzymes, and they detect both rat and human forms of the enzymes.

### *Cell lines*

The properties of the 20 SCLC cell lines used in these studies are summarised in Table I. The conditions for establishing and maintaining the cell lines have been described (Campling *et al.*, 1992). Twelve of the cell lines were established in this laboratory. The histologic features of the majority of these lines have been described (Campling *et al.*, 1992). Four of the lines (LD-T, MO-A, OS-A and SV-E) have not been previously described. Cell lines LD-T, MO-A, and OS-A are classical SCLC cell lines, and SV-E is an atypical SCLC. Cell lines NCI-H69, NCI-H128, and NCI-H209 were obtained from Dr J.D. Minna, NCI-Navy Medical Oncology Branch, National Institutes of Health, Bethesda, Maryland. H69AR is a doxorubicin-selected multidrug resistant variant of NCI-H69 which does not overexpress P-gp (Mirski *et al.*, 1987). Cell line Mar was obtained from Prof. A. Neville, Ludwig Institute for Cancer Research, London, UK. (Ibson *et al.*, 1987). SHP-77 was established by Fisher and Paulson, (1978), and was obtained from Dr J. Fogh, Sloan Kettering Institute for Cancer Research, New York. Cell lines RG-1 and MM-1 were obtained from Dr W.E.C. Bradley, Institute du Cancer de Montréal, and were characterised in this laboratory (Campling *et al.*, 1992).

All of the lines were grown in RPMI 1640 medium supplemented with hydrocortisone (10 nM), insulin (10 $\mu g\ ml^{-1}$), transferrin (10 $\mu g\ ml^{-1}$), estradiol (10 nM), and selenium (30 nM) (HITES medium) and 2.5% FBS. They were maintained in 5% $CO_2$ in a humidified atmosphere at 37°C. The treatment that the patients had received at the time that the cell lines were established is indicated in Table I. The response to further chemotherapy (if given and known) is also shown in this table.

### *Fresh SCLC tumour samples and early passage cell lines*

Fresh tumour samples were obtained from two patients. Sample No. 1 was pleural fluid from a patient who presented with extensive stage SCLC. He was treated initially with oral VP-16, and had a partial response. He then developed progressive disease despite further chemotherapy. A thoracentesis was performed to relieve dyspnea. The patient died shortly afterwards. Sample No. 2 was pericardial fluid from a patient who presented with extensive stage disease, and was treated initially with combination chemotherapy, consisting of cylcophosphamide, doxorubicin and vincristine, alternating with VP-16 and cisplatin. He had a partial response, but then developed progressive disease while on therapy. A pericardiocentesis was done to relieve cardiac tamponade. The patient improved temporarily, but then progressed on oral VP-16, and died.

For both of these fresh tumour samples, the fluid was centrifuged, and the cells were separated on a Ficoll-Hypaque gradient. The cells were put into tissue culture in HITES medium with 2.5% FBS and 50 $\mu l\ ml^{-1}$ gentamycin. Assays for GSH, GST, GSH Px and GSH Red were performed as outlined below. For patient sample No. 1, the assays were done on the day after the sample was received, and for sample No. 2, the assays were performed after the sample had been cultured for 5 days. Cytocentrifuge preparations were done to determine the proportion of tumour cells in each of the specimens.

Both of these tumour samples continued to grow in culture, and the results of GSH and related enzymes were repeated after several months in culture, and compared to the results on the original samples.

### *Drug sensitivity testing*

A modified MTT assay, as described previously (Campling *et al.*, 1991), was used to quantitate the response of the 20 cell lines to 16 different chemotherapeutic agents. The drugs

**Table I** SCLC cell lines

| *Cell line* | *Source* | *Prior treatment* | *Rx and response* | *Obtained from (reference)* |
|---|---|---|---|---|
| NCI-H69 | Pleural effusion | CMC-VAP | UK | J.D. Minna (Carney *et al.*, 1985; Carmichael *et al.*, 1988*b*) |
| H69AR | Multidrug resistant variant of NCI-H69 | Selected *in vitro* in doxorubicin | NA | S.P.C. Cole (Mirski *et al.*, 1987) |
| NCI-H128 | Pleural effusion | CMC-VAP | UK | J.D. Minna (Carney *et al.*, 1985; Carmichael *et al.*, 1988*b*) |
| NCI-H209 | Bone marrow | UT | UK | J.D. Minna (Carney *et al.*, 1985; Carmichael *et al.*, 1988*b*) |
| Mar | Primary tumour | S | UK | A. Neville (Ibson *et al.*, 1987) |
| SHP-77 | Primary tumour | S | UT-PD | J. Fogh (Fisher & Paulson, 1978) |
| AD-A | Needle aspirate of subcutaneous lesion | CA,VP/CP,RT | M-PD | This laboratory (Campling *et al.*, 1991) |
| BK-T | Primary tumour | S | CAV-NA | This laboratory (Campling *et al.*, 1991) |
| LG-T | Lymph node | UT | CAV,VP/CP-CR | This laboratory (Campling *et al.*, 1991) |
| HG-E | Pleural effusion | UT | UT-PD | This laboratory (Campling *et al.*, 1991) |
| JO-E | Pleural effusion | CAV,VP/CP,RT | UT-PD | This laboratory (Campling *et al.*, 1991) |
| WL-E | Pleural effusion | VP/CP,CAV | UT-PD | This laboratory (Campling *et al.*, 1991) |
| JN-M | Bone marrow | UT | CAV,VP/Carb-PD | This laboratory (Campling *et al.*, 1991) |
| SH-A | Needle aspirate of lymph node | CAV,VP/CP | CAV,VP/CP-PR | This laboratory (Campling *et al.*, 1991) |
| RG-1 | Pericardial effusion | CAV,VP/CP | UK-PD | W.E.C. Bradley (Campling *et al.*, 1991) |
| MM-1 | Pleural effusion | CAV,VP/CP | UK-PD | W.E.C. Bradley (Campling *et al.*, 1991) |
| LD-T | Primary tumour | S | CAV,VP/CP-NA | This laboratory (unpublished) |
| MO-A | Needle aspirate of lymph node | CAV,VP/CP | CAV,VP/CP-CR | This laboratory (unpublished) |
| OS-A | Needle aspirate of subcutaneous lesion | CAV,VP/CP | UT-PD | This laboratory (unpublished) |
| SV-E | Pleural effusion | CAV | VP/CP-PD | This laboratory (unpublished) |

The source of tumour cells from which the cell line was derived is indicated. Prior treatment indicates the therapy that the patient had received at the time that the cell line was established: UT – untreated; S – surgery; RT – radiotherapy; CMC-VAP – cyclophosphamide, methotrexate, CCNU, vincristine, doxorubicin, procarbazine; CAV – cyclosphosphamide, doxorubicin, vincristine; VP/CP-VP-16, cisplatin; carb – carboplatin; M – mitoxantrone.

Rx and response indicates the treatment that the patient received after the time that the cell line was established. The response to that treatment is also indicated: CR – complete response; PR – partial response; PD – progressive disease; NA – not assessable; UK – unknown.

tested included doxorubicin, amsacrine, carboplatin, cisplatin, 4-hydroperoxy-cyclophosphamide, daunomycin, epirubicin, melphalan, menogaril, mitomycin-C, mitoxantrone, nitrogen mustard, vinblastine, vincristine, VM-26, and VP-16. The response of the individual cell lines to each of the drugs tested was expressed as the area under the dose response curve (AUC) (Lam *et al.*, 1990).

*Assays for glutathione and related enzymes*

Cells from logarithmically growing cultures were harvested and used for these assays. Protein determinations were performed by the Lowry method as modified by Peterson (1977) for all assays except for the γ-GT assay, for which a one reagent method was used (BioRad, Richmond, CA).

The levels of total reduced and oxidised GSH were measured using a modification of the method of Tietze, (1969), with DTNB as a substrate. The assay was performed in microtiter plates, and the rate of colour formation at 410 nm was recorded. Results are expressed as μg GSH $mg^{-1}$ protein.

GST, GSH Red, and GSH Px assays were performed on cell cytosols prepared by sonicating $1-2 \times 10^7$ cells for three 15 s pulses with a 30 s pause between pulses, followed by centrifugation at 100,000 $g$ at 4°C for 20 min.

Total GST was measured using the method of Habig *et al.* (1974), with CDNB as a substrate. The rate of conjugation of CDNB with GSH was recorded at 340 nm. Results are expressed as nmol of CDNB reduced $min^{-1}$ $mg^{-1}$ protein.

GSH Px levels were measured by the method of Paglia and Valentine (1967) as modified by Reddy (1981) using hydrogen peroxide and cumene hydroperoxide as substrates. The assay follows the rate of loss of NADPH at 340 nm, and results were expressed as nmol NADPH consumed $min^{-1}$ $mg^{-1}$ protein.

GSH Red was measured using the method of Carlberg and Mannervik (1975), and results expressed as nmol NADPH consumed $min^{-1}$ $mg^{-1}$ protein.

γ-GT activity was measured on solubilised membrane preparations using a kit based on the method of Szasz (1969). Absorbance was followed at 405 nm, and results expressed as nmoles p-nitroaniline produced $min^{-1}$ $mg^{-1}$ protein.

*Immunodetection of GST isoenzymes*

Cytosolic preparations that had been assayed for GST activity were frozen at −20°C and used later for immunodetection of GST π, α, and μ. Cytosolic proteins (50 μg) were separated by electrophoresis on a 15% polyacrylamide gel (Laemmli, 1970). Purified human placental GST (No. isoenzyme) and rat liver GST (α and μ isoenzymes) were included as controls. Proteins were transferred overnight to Immobilon-P (Millipore, Mississauga, Ontario) at 100 mA according to the metnod of Towbin *et al.* (1979). The blots were then blocked with 5% skim milk in TBS for 1 h. Antibodies to GST π, μ, and α were diluted 1:5000 in TBS. The membranes were incubated with either pooled anti-GST-α and μ, or anti GST-π in 3% BSA/5% NGS/TBS for 1 h. The blots were incubated with alkaline phosphatase-conjugated goat anti-rabbit secondary antibody (1:8000) in 3% BSA/5% NGS/TBS. Immunodetection was performed using nitroblue tetrazolium and bromochloroindolyl phosphate as substrates (Cole *et al.*, 1990).

*Spearman correlation analysis*

Spearman rank correlation analysis (Armitage & Berry, 1987) was used to determine the relationship between the results of each of the GSH and related enzyme assays with the drug sensitivity results for the 16 different drugs. Cell line H69AR was omitted from this analysis, since it was the only cell line which had undergone *in vitro* selection for drug resistance. Thus, a total of 19 observations were included in the analysis (i.e. one AUC value for each of 19 cell lines – for some of the cell lines the AUC was the average of multiple observations, and for other cell lines it was a single measurement. The *P*-value indicating the significance of each individual correlation was also calculated (SAS Institute Inc., 1990). Scatter plots (96 of them) were produced to visualise the relationship between the variables. The Bonferroni multiple comparison method with the Fisher z-transformation of the correlation coefficients was applied to determine significant correlations at the overall 0.05 level of significance (Morrison, 1976).

*Correlation with treatment status*

To correlate the results of GSH and related enzymes with the treatment status of the patients from whom the cell lines were derived, the cell lines were classified as 'treated' or 'untreated' with chemotherapy, on the basis of the clinical information in Table I. The mean values for cell lines from treated patients were compared to the mean values for cell lines from untreated patients. Because of the skewed distribution of the data, the Wilcoxan rank sum (or Mann-Whitney U) test was used to make this comparison (Armitage & Berry, 1987; SAS Institute Inc., 1990).

### Results

*Drug sensitivity testing*

The results of chemosensitivity testing for 16 of the cell lines (NCI-H69, H69AR, NCI-H128, NCI-H209, Mar, SHP-77, AD-A, BK-T, LG-T, HG-E, JO-E, WL-E, JN-M, SH-A, RG-1, and MM-1) have been published previously (Campling *et al.*, 1991). The results with the remaining four cell lines (LD-T, MO-A, OS-A, and SV-E) are summarised in Table II. The results are expressed as area under the dose response curve, calculated using the trapezoidal method as described (Lam *et al.*, 1990). It can be seen that these four cell lines are quite similar in terms of response to the various drugs. The previously reported 16 cell lines represent a wider range of drug responsiveness.

*Glutathione and related enzyme levels*

The levels of GSH, GST, GSH Px, GSH Red and γ-GT in the 20 cell lines are shown in Table III. It can be seen that each of the cell lines had a characteristic and reproducible profile of GSH and associated enzyme levels. The GSH levels ranged from 0.35 to 7.69 μg $mg^{-1}$ protein in the SCLC lines. GST levels varied from 4.83 to 248 nmol $min^{-1}$ $mg^{-1}$ protein. GSH Px ranged from 0 to 49.3 nmol $min^{-1}$ $mg^{-1}$ protein

**Table II** Chemosensitivity results

| | Cell line | | | |
|---|---|---|---|---|
| *Drug* | *LD-T* | *MO-A* | *OS-A* | *SV-E* |
| Doxorubicin | 37.3 | 29.4 | 34.0 | 67.8 |
| Amsacrine | 9.8 | 11.1 | 18.8 | 19.0 |
| Carboplatin | 54.0 | 80.1 | 98.2 | 89.2 |
| Cisplatin | 43.0 | 31.6 | 67.9 | 55.6 |
| 4H-cyclophosphamide | 57.9 | 52.9 | 61.9 | 88.3 |
| Daunomycin | 9.3 | 5.8 | 10.7 | 13.8 |
| Epirubicin | 23.7 | 10.8 | 28.7 | 29.8 |
| Melphalan | 28.4 | 32.5 | 62.2 | 63.2 |
| Menogaril | 10.4 | 14.2 | 16.2 | 13.8 |
| Mitomycin C | 29.8 | 20.8 | 37.7 | 30.9 |
| Mitoxantrone | 19.9 | 17.6 | 22.7 | 40.5 |
| Nitrogen mustard | 26.3 | 17.9 | 45.3 | 33.1 |
| Vinblastine | 48.9 | 35.9 | 60.3 | 47.4 |
| Vincristine | 65.8 | 67.5 | 74.7 | 95.1 |
| VM-26 | 31.2 | 28.6 | 32.4 | 13.6 |
| VP-16 | 80.2 | 58.6 | 73.1 | 70.5 |

The chemosensitivity results for 16 of the cell have been published (Campling *et al.*, 1991). The results for the remaining four cell lines are presented here. Results are expressed as area under the dose response curve (Lam *et al.*, 1990).

with $H_2O_2$ and from 1.47 to 47.7 nmol $min^{-1} mg^{-1}$ protein with cumene hydroperoxide as a substrate. GSH Red levels varied from 13.0 to 115 nmol $min^{-1} mg^{-1}$ protein, and γ-GT ranged from 0 to 9.1 nmol $min^{-1} mg^{-1}$ protein.

### *Spearman correlation analysis*

The results of the correlation analysis are shown in Table IV. The six GSH related measurements were correlated with sensitivities to 16 different drugs, giving a total of 96 different pairs of correlations. Table IV shows that there were four correlation coefficients with $P$ values $\leqslant 0.05$. This is no greater than would be expected by chance alone. The highest correlation coefficient was between response to 4-hydroperoxy-cyclophosphamide and GSH levels ($r = 0.64$, $P = 0.004$). Other correlations with $P$ values $\leqslant 0.05$ included a negative correlation with m-amsacrine response and GST levels ($r = 0.49$, $P = 0.03$), and a positive correlation between

**Table III** GSH and related enzymes in SCLC cell lines

| *Cell line* | *GSH* | *GST* | *GSH Px* $H_2O_2$ | *GSH Px* *Cumene* | *GSH Red* | *γ-GT* |
|---|---|---|---|---|---|---|
| NCI-H69 | 3.89 ± 1.53 | 163 ± 18 | 7.1 ± 2.7 | 10.0 ± 2.8 | 34.5 ± 2.6 | 0.39 ± 0.20 |
| H69AR | 0.35 ± 0.28 | 185 ± 11 | 10.0 ± 5.8 | 9.98 ± 2.9 | 37.7 ± 11.7 | 5.07 ± 1.09 |
| NCI-H128 | 0.96 ± 0.32 | 19.8 ± 3.4 | 14.8 ± 4.9 | 14.7 ± 4.7 | 52.4 ± 8.8 | 0.37 ± .24 |
| HCI-H209 | 4.28 ± 2.25 | 170 ± 25 | 16.7 ± 4.7 | 22.0 ± 2.3 | 40.6 ± 1.9 | 0.12 ± .12 |
| Mar | 1.28 ± 0.56 | 89.2 ± 22.6 | 5.0 ± 2.3 | 5.2 ± 3.9 | 44.9 ± 9.6 | 0.68 ± .02 |
| SHP-77 | 5.52 ± 3.02 | 92.1 ± 21.5 | 28.9 ± 15.7 | 34.4 ± 10.5 | 115 ± 11 | 9.10 ± 1.96 |
| AD-A | 2.46 ± 0.79 | 42.9 ± 8.7 | 5.88 ± 1.17 | 6.83 ± .85 | 57.2 ± 18 | 0.036 ± .042 |
| BK-T | 3.16 ± 1.33 | 9.79 ± 2.48 | 18.4 ± 4.8 | 19.8 ± 12.9 | 44.5 ± 7.2 | 0.02 ± .03 |
| LG-T | 1.57 ± 0.63 | 248 ± 59 | 16.6 ± 7.4 | 8.9 ± 1.6 | 17.6 ± 0.8 | 0.23 ± .25 |
| HG-E | 2.22 ± 0.97 | 191 ± 35 | 0 ± 0 | 1.47 ± 1.41 | 21.1 ± 5.0 | 0.18 ± .15 |
| JO-E | 2.18 ± 1.75 | 11.1 ± 4.8 | 11.0 ± 3.6 | 8.03 ± 5.57 | 56.9 ± 5.4 | 0.31 ± .14 |
| WL-E | 3.06 ± 1.66 | 172 ± 22 | 49.3 ± 1.8 | 37.8 ± 11.3 | 13.0 ± 1.7 | 0.24 ± .12 |
| JN-M | 1.49 ± 0.77 | 104 ± 24 | 12.7 ± 6.9 | 13.4 ± 3.7 | 37.8 ± 3.5 | 0.36 ± .37 |
| SH-A | 2.17 ± 0.75 | 39.6 ± 29.6 | 10.4 ± 2.4 | 7.3 ± 7.5 | 37.3 ± 5.0 | 0.18 ± .30 |
| RG-1 | 2.97 ± 1.59 | 5.66 ± 3.71 | 17.5 ± 6.2 | 17.3 ± 3.4 | 15.0 ± 2.6 | 0.19 ± .08 |
| MM-1 | 3.43 ± 2.09 | 71.7 ± 4.7 | 19.2 ± 4.7 | 19.1 ± 3.6 | 21.0 ± 4.2 | 0 ± 0 |
| LD-T | 4.81 ± 2.53 | 64.4 ± 3.7 | 5.7 ± 2.2 | 5.05 ± 1.80 | 50.0 ± 4.0 | 0.28 ± .28 |
| MO-A | 3.21 ± 1.62 | 115 ± 29 | 9.57 ± 2.43 | 7.69 ± 2.84 | 32.3 ± 7.0 | 0.56 ± .49 |
| OS-A | 7.69 ± 2.04 | 4.83 ± .71 | 24.0 ± 2.1 | 18.6 ± 7.0 | 35.7 ± 4.7 | 0.29 ± .04 |
| SV-E | 7.18 ± 3.04 | 241 ± 35 | 36.1 ± 9.1 | 47.7 ± 21.4 | 108 ± 5.0 | 7.55 ± 1.11 |

Levels of GSH, GST, GSH Px, GSH Red and γ-GT were determined for 20 cell lines. Assays were performed at least three times on each cell line, and standard deviations are indicated. The units for GSH are μg $ml^{-1}$ protein. All enzyme levels are expressed as nmol $min^{-1} mg^{-1}$ protein.

**Table IV** Spearman correlation analysis

| *Drug* | *GSH* | *GST* | *GSH Px* $H_2O_2$ | *GSH Px* *Cumene* | *GSH Red* | *γ-GT* |
|---|---|---|---|---|---|---|
| Doxorubicin | 0.12 | 0.17 | 0.09 | 0.03 | −0.21 | 0.02 |
| | (0.62) | (0.48) | (0.70) | (0.91) | (0.38) | (0.92) |
| Amsacrine | −0.01 | **−0.49** | **0.60** | **0.50** | −0.07 | −0.09 |
| | (0.95) | **(0.03)** | **(0.006)** | **(0.03)** | (0.78) | (0.71) |
| Carboplatin | 0.03 | −0.22 | 0.14 | −0.01 | −0.15 | 0.24 |
| | (0.91) | (0.36) | (0.56) | (0.96) | (0.54) | (0.32) |
| Cisplatin | 0.17 | −0.24 | 0.26 | 0.07 | −0.02 | 0.14 |
| | (0.51) | (0.34) | (0.30) | (0.78) | (0.95) | (0.58) |
| 4H-Cyclophosphamide | **0.64** | −0.05 | 0.25 | 0.10 | 0.03 | 0.31 |
| | **(0.004)** | (0.84) | (0.32) | (0.69) | (0.92) | (0.20) |
| Daunomycin | −0.25 | −0.26 | 0.24 | 0.03 | −0.13 | −0.14 |
| | (0.31) | (0.28) | (0.32) | (0.91) | (0.61) | (0.57) |
| Epirubicin | 0.16 | 0.11 | 0.20 | 0.12 | 0.11 | 0.21 |
| | (0.52) | (0.64) | (0.40) | (0.64) | (0.65) | (0.40) |
| Melphalan | 0.24 | 0.03 | 0.24 | 0.07 | −0.04 | 0.31 |
| | (0.32) | (0.90) | (0.32) | (0.76) | (0.88) | (0.19) |
| Menogaril | 0.03 | −0.10 | 0.27 | 0.05 | −0.22 | 0.23 |
| | (0.91) | (0.69) | (0.26) | (0.83) | (0.37) | (0.34) |
| Mitomycin C | 0.09 | −0.29 | 0.26 | 0.05 | 0.07 | 0.09 |
| | (0.71) | (0.23) | (0.28) | (0.84) | (0.77) | (0.71) |
| Mitoxantrone | −0.12 | 0.07 | 0.22 | 0.12 | −0.18 | 0.01 |
| | (0.63) | (0.76) | (0.35) | (0.64) | (0.47) | (0.98) |
| Nitrogen mustard | 0.24 | 0.00 | 0.01 | −0.12 | −0.03 | 0.11 |
| | (0.32) | (0.99) | (0.97) | (0.62) | (0.90) | (0.66) |
| Vinblastine | 0.08 | −0.07 | 0.06 | −0.09 | −0.14 | 0.22 |
| | (0.76) | (0.76) | (0.79) | (0.70) | (0.58) | (0.37) |
| Vincristine | −0.14 | 0.03 | −0.08 | −0.24 | −0.08 | 0.06 |
| | (0.57) | (0.89) | (0.75) | (0.32) | (0.75) | (0.79) |
| VM-26 | −0.09 | −0.40 | 0.07 | −0.20 | −0.24 | −0.14 |
| | (0.73) | (0.10) | (0.79) | (0.42) | (0.34) | (0.59) |
| VP-16 | 0.20 | −0.12 | −0.10 | −0.24 | −0.04 | −0.13 |
| | (0.41) | (0.61) | (0.69) | (0.33) | (0.87) | (0.60) |

The levels of GSH and related enzymes were correlated with the drug sensitivity of the cell lines to 16 different drugs. The correlation coefficients are indicated. The statistical significance of these correlations ($P$ values) is shown in brackets below the correlation coefficients. Correlation coefficients with $P$ values $\leqslant 0.05$ are in boldface.

m-amsacrine response and levels of GSH Px, using hydrogen peroxide ($r = 0.60$, $P = 0.006$) and cumene hydroperoxide ($r = 0.50$, $P = 0.03$) as substrates. Scatter plots corresponding to the 96 correlations made in this study did not show any apparent non-linear relationships between the variables.

With a total of 96 correlations to be considered in this study, correlation coefficients exceeding 0.66 are considered significant using the Bonferroni method. The conclusions of the Bonferroni simultaneous tests lead to a more cautious assessment of the strengths of the relationships between the assays and the drug sensitivity results. However, the Bonferroni approach may be excessively conservative, and thus the *P* value for each individual correlation is also given in Table IV.

We have also correlated levels of these various enzymes with themselves (data not shown). The total GSH levels correlated with GSH Px activity measured with both $H_2O_2$ ($r = 0.49$, $P = 0.02$) and cumene hydroperoxide ($r = 0.42$, $P = 0.05$) as substrates. In addition, GSH Red correlated with γ-GT levels ($r = 0.49$, $P = 0.02$). The GSH Px levels using the two different substrates, $H_2O_2$ and cumene hydroperoxide, were highly correlated ($r = 0.90$, $P = 0.0001$).

### *Correlation with treatment status*

The GSH and associated enzyme levels in the cell lines derived from treated patients were compared to those derived from untreated patients. There were no significant differences between the two groups of cell lines (data not shown).

### *Fresh SCLC tumour samples and early passage cell lines*

Both of the fresh tumour samples consisted of more than 90% tumour cells. The results of assays for GSH and related enzymes are shown in Table V. It can be seen that the values obtained are in the same range as for the SCLC cell lines.

The assays were repeated after the samples had been maintained *in vitro* for several months. It can be seen from Table V that the values for many of the assays were not stable after prolonged tissue culture. For example, the initial GST level for sample No. 1 was 83.5 nmol $min^{-1}$ $mg^{-1}$ protein, but after several months, this level had declined to 20.7 nmol $min^{-1}$ $mg^{-1}$ protein. On the other hand, for sample No. 2, the level of GSH Red increased from 7.74 nmol $min^{-1}$ $mg^{-1}$ protein initially, to 25.6 nmol $min^{-1}$ $mg^{-1}$ protein after *in vitro* culture.

### *Immunodetection of GST isoenzymes*

Immunoblots using antibodies to three subclasses of GST (α, μ and π) were performed on 15 cell lines. The remaining lines had levels of GST that were too low for detection on immunoblots (cell lines OS-A, JO-E and RG-1), or insufficient amounts of cell lysate available at the time (cell lines Mar, SH-A and MO-A).

The results are shown in Figure 1. The antibody to GST π detected the GST isoenzyme(s) present in human placenta, but not rat liver, and the antibodies to GST α and μ detected GST isoenzymes in rat liver, but not human placenta. It can be seen that the π isoenzyme of GST was present in these 15 cell lines. The α and μ isoenzymes were not detectable in any of these unpurified samples (data not shown).

In addition to the SCLC lines, a cell line BH-E is shown in Figure 1. This line was derived from a recurrent pleural effusion in a patient who was originally diagnosed and treated as SCLC. However, the histology on the cell line derived from this patient showed adenocarcinoma. This cell line was not included in the correlation analysis.

### Discussion

Despite the fact that many groups have studied the role of alterations in glutathione metabolism in drug resistant SCLC, the importance of these mechanisms of resistance in this tumour has not been clarified. In this study, very few

**Figure 1** Immunoblots using antibody to GST-π. Human placental GST (GST π) (0.5 μg/lane) is included as a positive control and purified rat liver GST (GST α and μ) (0.5 μg/lane) as a negative control on the blots using the antibody to GST π. Cytosolic proteins (50 μg) were separated by electrophoresis on a 15% polyacrylamide gel. Proteins were transferred to Immobilon-P, and immunodetection of GST π was performed as described in the text.

**Table V** GSH and related enzymes in fresh tumour samples and early passage cell lines

| Sample | GSH | GST | GSH Px $H_2O_2$ | GSH Px Cumene | GSH Red |
|---|---|---|---|---|---|
| No. 1 (original) | 7.28 | 83.5 | 36.4 | 43.5 | 86.4 |
| (Early passage) | 1.95 | 20.7 | 10.3 | 15.3 | 74.3 |
| No. 2 (original) | 0.96 | 40.5 | 0 | 5.27 | 7.74 |
| (Early passage) | 0.70 | 44.4 | 0.66 | 4.7 | 25.6 |

The units for GSH are μg $ml^{-1}$ protein. All enzyme levels are expressed as nmol $min^{-1}$ $mg^{-1}$ protein.

significant correlations were found between levels of GSH or its related enzymes and drug sensitivity of these human SCLC cell lines. Furthermore, there were no differences in levels in cell lines derived from treated patients compared to those derived from untreated patients. Thus, our results provide support for the idea that GSH related detoxification mechanisms do not play a major role in drug resistance in SCLC.

In this report, we have examined larger numbers of SCLC lines than previously studied by other investigators. The drug sensitivity profiles of these lines have been extensively characterised, and they display a wide range of drug sensitivity (Campling *et al.*, 1991). Furthermore, the treatment histories of the patients from whom these lines were obtained are well documented, and they are known to represent a spectrum of clinically drug sensitive and drug resistant tumours. Thus, our results may reflect the clinical situation.

The GST's have been intensively investigated as potential mediators of drug resistance. These enzymes catalyse the conjugation of GSH to electrophilic substances, thus rendering them more water soluble. The observation by Batist *et al.* (1986) that a multidrug resistant human breast cancer cell line overexpressed the π isoenzyme of GST led to increased interest in this isoenzyme as a potential marker of drug resistance. Subsequently, numerous groups have found increased expression of GST π in cell lines and clinical samples which are resistant to alkylating agents (Buller *et al.*, 1987; Gupta *et al.*, 1989; Armstrong et al., 1992), nitrosoureas (Ali-Osman *et al.*, 1990), platinum compounds (Teicher *et al.*, 1987; Nakagawa *et al.*, 1988; Saburi *et al.*, 1989), and natural product drugs (Whelan *et al.*, 1989; Peters & Roelofs, 1992; Singh *et al.*, 1989). However, the functional role of GST π in drug resistance has been questioned, since many multidrug resistant cell lines do not overexpress this isoenzyme (Yusa *et al.*, 1988). Furthermore, a multidrug resistant variant of the breast cancer cell line, MCF-7, which overexpresses GST π, retained elevated levels of this enzyme when it reverted to a drug sensitive phenotype (Yusa *et al.*, 1988).

One of the most direct approaches to resolving the question of whether increased expression of GST π alone confers drug resistance is to transfect the gene into drug sensitive cells, and examine the drug response of the transfectants. A number of groups have taken this approach, with variable results. Some have found no changes in drug sensitivity of the transfectants, despite increased levels of GST π activity (Moscow *et al.*, 1989; Fairchild *et al.*, 1990). Others have found GST π transfected cells to be resistant to doxorubicin, whereas the effect on sensitivity to alkylating agents has been variable (Nakagawa *et al.*, 1990; Puchalsky & Fahl, 1990; Black *et al.*, 1989). In general, the levels of resistance detected have been low.

One of the largest studies of the role of GSH metabolism as a determinant of drug sensitivity in lung cancer was that of Carmichael *et al.* (1988*c*), which examined 30 different SCLC and NSCLC cell lines. Significant differences were found between the results for the SCLC compared to the NSCLC lines. They speculated that these alterations could account for the differences in drug sensitivity between these two tumour types. However, a reanalysis of this data by Hosking *et al.* (1990) showed no correlation between GSH levels and doxorubicin response in these cell lines. Furthermore, no differences were found between results for cell lines from previously untreated and previously treated patients. Our results are in accordance with these findings.

We found that the only detectable GST in our cell lines was the π isoenzyme. Others have found that GST π is the predominant transferase in normal lung, and is overexpressed in lung tumours (Howie *et al.*, 1990; Awasthi *et al.*, 1987; Carmichael *et al.*, 1988*a*).

Although our data do not show significant correlations between drug sensitivity and alterations in GSH metabolism, it is possible that we may have underestimated the importance of these metabolic pathways. In some experimental systems, increased capacity for glutathione synthesis in response to cytotoxic drugs has been demonstrated (Ahmad *et al.*, 1987; Lee *et al.*, 1989; Meijer *et al.*, 1990). Such changes may not be detected by static measurements of levels of GSH and associated enzymes, as carried out in this study.

It is also possible that changes in subcellular localisation of GSH and related enzymes may be involved in mediating drug resistance in SCLC. For example, Lutzky *et al.* (1989) have detected altered subcellular distribution of GSH and GST in a multidrug resistant variant of the leukaemia cell line, HL60, using a fluorescent probe, monochlorobimane, which is conjugated to GSH by GST. Since our measurements were done on whole cell lysates, we would not have detected such alterations.

Another concern is that the GSH and related enzyme levels, and/or the drug sensitivity profiles of the cell lines may not reflect the levels from the original tumour samples from which the lines were derived. In a previous report we found that the drug sensitivity of cell lines from untreated patients was greater than that of lines from treated patients, although the results were statistically significant for only two of the drugs (Campling *et al.*, 1991). Some of these cell lines have been in continuous culture for several years. We have not seen any major change in drug sensitivity profiles of these lines over the years. However, it is possible that GSH and related enzyme levels may have altered. In the case of one cell line (NCI-H69), we found that the GST level increased over a two year period (Cole *et al.*, 1991).

For this reason, we examined fresh tumour samples and early passage cell lines from two patients with SCLC. Both of these patients had developed recurrent disease while on chemotherapy, and were considered clinically drug resistant. The results from the early passage cell lines indicate that some of the measurements were not stable after *in vitro* culture. This raises the important concern that some features of human tumour cell lines may not continue to represent the tumours from which they were derived. Although both of these patients were clinically drug resistant, the levels of GSH and related enzymes in tumour samples and early passage cell lines derived from them were not particularly high when compared to the panel of cell lines.

An understanding of clinically relevant resistance mechanisms in SCLC (as well as other tumour types) could lead to strategies for circumventing drug resistance. Early phase clinical trials of agents such as buthionine sulfoximine, an inhibitor of GSH synthesis (Griffith, 1982), or ethacrynic acid, an inhibitor of GST, are currently underway (O'Dwyer *et al.*, 1991; 1992). These agents may prove to be beneficial in those tumour types in which alterations in GSH metabolism are clinically important. It appears that ovarian cancer may fall into this category (Andrews *et al.*, 1985; Ozols *et al.*, 1987; Mistry *et al.*, 1991), but from the data presented here, it appears that SCLC does not. It is quite likely that different tumour types may invoke different cellular protection mechanisms in response to chemotherapy.

The acquisition of resistance to multiple chemotherapeutic agents continues to be the major impediment to cure of SCLC. Despite intensive efforts of many investigators, the solution to this problem has remained elusive. Evidence from many laboratories suggests that P-gp is not a major factor. The data presented here indicate that alterations in GSH metabolism do not likely play a major role.

Attention should now be directed toward investigating other potential mechanisms of multidrug resistance in SCLC, and determining their clinical importance. There is increasing evidence for involvement of the nuclear enzyme, topoisomerase II, in multidrug resistance in SCLC (Long *et al.*, 1986; Zijlstra *et al.*, 1987; de Jong *et al.*, 1990; Binashi *et al.*, 1990; Minato *et al.*, 1990; Cole *et al.*, 1991; Kasahara *et al.*, 1992; Giaccone *et al.*, 1992). This enzyme is the intranuclear target of many of the drugs which are part of the 'multidrug resistance phenotype'. However, changes in topoisomerase II cannot explain the entire problem of drug resistance in SCLC, since they would not account for resistance to the vinca alkaloids, alkylating agents, or platinum compounds which do not interact with this DNA unknotting enzyme. Thus, drug resistance in this disease may be multifactorial (Cole, 1992).

Recently, a number of groups have reported changes in subcellular distribution of drugs in multidrug resistant cell lines which do not overexpress P-gp (Gervasoni *et al.*, 1991; Schuurhuis *et al.*, 1991; Marquardt & Center, 1992). These findings suggest the existence of other transport protein(s) apart from P-gp, which may act by sequestering drugs away from their intracellular targets. The recent isolation of a cDNA encoding a novel ATP-binding cassette transporter protein which is overexpressed in the H69AR cell line may shed light on some of these observations (Cole *et al.*, 1992). Further investigation of the role of this transporter in clinically acquired drug resistance in SCLC is warranted.

The authors thank Drs K. Tew and M. Clapper for providing the GST antibodies, and for guidance in the immunoblotting experiments.

This work was supported by the Medical Research Council of Canada, and the Ontario Cancer Treatment and Research Foundation.

**Abbreviations:** SCLC, small cell lung cancer; GSH, glutathione; GST, glutathione S-transferase; GSH Red, glutathione reductase; GSH Px, glutathione peroxidase; γ-GT, γ-glutamyltranspeptidase; FBS, foetal bovine serum; MTT, 3-[4,5-dimethylthiazol-2-yl]-2,5-diphenyltetrazolium bromide; CDNB, 1-chloro-2,4-dinitrobenzene; DTNB, 5,5′-dithiobis(2-nitrobenzoic acid); BSA, bovine serum albumin; TBS, Tris buffered saline; NGS, normal goat serum.

### References

AHMAD, S., OKINE, L., LE, B., NAJARIAN, P. & VISTICA, D.T. (1987). Elevation of glutathione in phenylalanine mustard-resistant murine L1210 leukemia cells. *J. Biol. Chem.*, **262,** 15048–15053.

ALI-OSMAN, F., STEIN, D.E. & RENWICK, A. (1990). Glutathione content and glutathione S-transferase expression in 1,3-bis(2-chloroethyl)-1-nitrosourea-resistant human malignant astrocytoma cell lines. *Cancer Res.*, **50,** 6976–6980.

ANDREWS, P.A., MURPHY, M.P. & HOWELL, S.B. (1985). Differential potentiation of alkylating and platinating agent cytotoxicity in human ovarian carcinoma cells by glutathione depletion. *Cancer Res.*, **45,** 6250–6253.

ARMITAGE, P. & BERRY, G. (1987). *Statistical Methods in Medical Research*, 2nd Edition. Oxford: Blackwell Scientific Publ, pp. 411–420.

ARMSTRONG, D.K., GORDON, G.B., HILTON, J., STREEPER, R.T., COLVIN, O.M. & DAVIDSON, N.E. (1992). Hepsulfam sensitivity in human breast cancer cell lines: The role of glutathione and glutathione S-transferase in resistance. *Cancer Res.*, **52,** 1416–1421.

AWASTHI, Y.C., SINGH, S.V., AHMAD, H. & MOLLER, P.C. (1987). Immunocytochemical evidence for the expression of GST1, GST2, GST3 gene loci for glutathione S-transferase in human lung. *Lung*, **165,** 323–332.

BATIST, G., TULPULE, A., SINHA, B., KATAOKI, A.G., MYERS, C.E. & COWAN, K.H. (1986). Overexpression of a novel anionic glutathione transferase in multi-drug resistant human breast cancer cells. *J. Biol. Chem.*, **261,** 15544–15549.

BINASHI, M., CAPRANICO, G., DE ISABELLA, P., MARIANI, M., SUPINO, R., TINELLI, S & ZUNINO, F. (1990). Comparison of DNA cleavage induced by etoposide and doxorubicin in two small-cell lung cancer lines with different sensitivities to topoisomerase II inhibitors. *Int. J. Cancer*, **45,** 347–352.

BLACK, S.M., BEGGS, J.D., HAYES, J.D., BARTOSZEK, A., MURAMATSU, M., SAKAI, M. & WOLF, C.R. (1989). Expression of human glutathione S-transferases in *Saccharomyces cerevisiae* confers resistance to anticancer drugs adriamycin and chlorambucil. *Biochem. J.*, **268,** 309–315.

BRADLEY, G., JURANKA, P. & LING, V. (1988). Mechanism of multidrug resistance. *Biochim. Biophys. Acta*, **948,** 87–128.

BULLER, A.C., CLAPPER, M.L. & TEW, K.D. (1987). Glutathione-S-transferases in nitrogen mustard-resistant and -sensitive cell lines. *Mol. Pharmacol.*, **31,** 575–578.

CAMPLING, B.G., HAWORTH, A.C., BAKER, H.M., GREER, D.L., HOLDEN, J.J.A., BRADLEY, W.E.C., PYM, J. & DEXTER, D.F. (1992). Establishment and characterization of a panel of human lung cancer cell lines. *Cancer*, **69,** 2064–2074.

CAMPLING, B.G., PYM, J., BAKER, H.M., COLE, S.P.C. & LAM, Y.-M. (1991). Chemosensitivity testing of small cell lung cancer using the MTT assay. *Br. J. Cancer*, **63,** 75–83.

CARLBERG, I. & MANNERVIK, B. (1975). Purification and characterization of the flavoenzyme glutathione reductase from rat liver. *J. Biol. Chem.*, **250,** 5475–5480.

CARMICHAEL, J., FORRESTER, L.M., LEWIS, A.D., HAYES, J.D., HAYES, P.C. & WOLF, C.R. (1988*a*). Glutathione S-transferase isoenzymes and glutathione peroxidase activity in normal and tumour samples from human lung. *Carcinogenesis*, **9,** 1617–1621.

CARMICHAEL, J., MITCHELL, J.B., DEGRAFF, W.G., GAMSON, J., GAZDAR, A.F., JOHNSON, B.E., GLATSTEIN, E. & MINNA, J.D. (1988*b*). Chemosensitivity testing of human lung cancer cell lines using the MTT assay. *Br. J. Cancer*, **57,** 540–547.

CARMICHAEL, J., MITCHELL, J.B., FRIEDMAN, N., GAZDAR, A.F. & RUSSO, A. (1988*c*). Glutathione and related enzyme activity in human lung cancer cell lines. *Br. J. Cancer*, **58,** 437–440.

CARNEY, D.N., GAZDAR, A.F., BEPLER, G., GUCCION, J.G., MARANGOS, P.J., MOODY, T.W., ZWEIG, M.H. & MINNA, J.D. (1985). Establishment and identification of small cell lung cancer cell lines having classic and variant features. *Cancer Res.*, **45,** 2913–2923.

COHEN, M.H. & MATTHEWS, M.J. (1978). Small cell bronchogenic carcinoma. A distinct clinicopathologic entity. *Semin. Oncol.*, **5,** 234–243.

COLE, S.P.C. (1992). The 1991 Merck Frosst Award: Multidrug resistance in small cell lung cancer. *Can. J. Physiol. Pharmacol.*, **70,** 313–329.

COLE, S.P.C., BHARDWAJ, G., GERLACH, J.H., MACKIE, J.E., GRANT, C.E., ALMQUIST, K.C., STEWART, A.J., KURZ, E.U., DUNCAN, A.M.V. & DEELEY, R.G. (1992). Overexpression of a transporter gene in multidrug-resistant human lung cancer cell line. *Science*, **258,** 1650–1654.

COLE, S.P.C., CHANDA, E.R., DICKE, F.P., GERLACH, J.H. & MIRSKI, S.E.L. (1991). Non-P-glycoprotein mediated multidrug resistance in a small cell lung cancer cell line: Evidence for decreased susceptibility to drug-induced DNA damage and reduced levels of topoisomerase II. *Cancer Res.*, **51,** 3345–3352.

COLE, S.P.C., DOWNES, H.F., MIRSKI, S.E.L. & CLEMENTS, D.J. (1990). Alterations in glutathione and glutathione-related enzymes in a multidrug resistant small cell lung cancer cell line. *Molec. Pharmacol.*, **37,** 192–197.

DE JONG, S., ZIJLSTRA, J.G., DE VRIES, E.G.E. & MULDER, N.H. (1990). Reduced DNA topoisomerase II activity and drug-induced DNA cleavage activity in an adriamycin-resistant human small cell lung carcinoma cell line. *Cancer Res.*, **50,** 304–309.

FAIRCHILD, C.R., MOSCOW, J.A., O'BRIEN, E.O. & COWAN, K.H. (1990). Multidrug resistance in cells transfected with human genes encoding a variant P-glycoprotein and glutathione S-transferase π. *Molec. Pharmacol.*, **37,** 801–809.

FISHER, E.R. & PAULSON, J.D. (1978). A new *in vitro* cell line established from human large cell variant of oat cell lung cancer. *Cancer Res.*, **38,** 3830–3835.

GERVASONI, J.E., FIELDS, S.Z., KRISHNA, S., BAKER, M.A., ROSADO, M., THURAISAMY, K., HINDENBURG, A.A. & TAUB, R.N. (1991). Subcellular distribution of daunorubicin in P-glycoprotein-positive and -negative drug-resistant cell lines using laser-assisted confocal microscopy. *Cancer Res.*, **51,** 4955–4963.

GIACCONE, G., GAZDAR, A.F., BECK, H., ZUNINO, F. & CAPRANICO, G. (1992). Multidrug sensitivity phenotype of human lung cancer cells associated with topoisomerase II expression. *Cancer Res.*, **52,** 1666–1674.

GRIFFITH, O.W. (1982). Mechanism of action, metabolism, and toxicity of buthionine sulfoximine and its higher homologs, potent inhibitors of glutathione synthesis. *J. Biol. Chem.*, **257,** 13704–13712.

GUPTA, V., SINGH, S.V., AHMAD, H., MEDH, R.D. & AWASTHI, J. (1989). Glutathione and glutathione-S-transferases in a human plasmacytoma cell line resistant to melphalan. *Biochem. Pharmacol.*, **38,** 1993–2000.

HABIG, W.H., PABST, M.J. & JAKOBY, W.B. (1974). Glutathione S-transferases. *J. Biol. Chem.*, **249,** 7130–7139.

HOSKING, L.K., WHELAN, R.D.H., SHELLARD, S.A., BEDFROD, P. & HILL, B.T. (1990). An evaluation of the role of glutathione and its associated enzymes in the expression of differential sensitivities to antitumour agents shown by a range of human tumour cell lines. *Biochem. Pharmacol.*, **40**, 1833–1842.

HOSPERS, G.A.P., MULDER, N.H., DEJONG, B., DELEY, L., UGES, D.R.A., FICHTINGER-SCHEPMNAN, A.M.J., SCHEPER, R.J. & DEVRIES, E.G.E. (1988). Characterization of a human small cell lung carcinoma cell line with acquired resistance to cis-diamminedichloroplatinum (II) *in vitro*. *Cancer Res.*, **48**, 6803–6807.

HOWIE, A.F., FORRESTER, L.M., GLANCEY, M.J., SCHLAGER, J.J., POWIS, G., BECKETT, G.J., HAYES, J.D. & WOLF, C.R. (1990). Glutathione S-transferase and glutathione peroxidase expression in normal and tumour human tissues. *Carcinogenesis*, **11**, 451–458.

IBSON, J.M., WATERS, J.J., TWENTYMAN, P.R., BLEEHEN, N.M. & RABBITTS, P.H. (1987). Oncogene amplification and chromosomal abnormalities in small cell lung cancer. *J. Cell Biochem.*, **33**, 267–288.

KASAHARA, K., FUJIWARA, Y., SUGIMOTO, Y., NISHIO, K., TAMURA, T., MATSUDA, T. & SAIJO, N. (1992). Determinants of response to the DNA topoisomerase II inhibitors doxorubicin and etoposide in human lung cancer cell lines. *J. Natl Cancer Inst.*, **84**, 113–118.

KRAMER, R.A., ZAKHER, J. & KIM, G. (1988). Role of the glutathione redox cycle in acquired and *de novo* multidrug resistance. *Science*, **241**, 694–697.

LAEMMLI, U.K. (1970). Cleavage of structural proteins during the assembly of the head of bacteriophage T4. *Nature*, **227**, 680–685.

LAI, S.-L., GOLDSTEIN, L.J., GOTTESMAN, M.M., PASTAN, I., TSAI, C.-M., JOHNSON, B.E., MULSHINE, J.L., IHDE, D.C., KAYSER, K. & GAZDAR, A.F. (1989). MDR1 gene expression in lung cancer. *J. Natl Cancer Inst.*, **81**, 1144–1150.

LAM, Y.-M., PYM, J. & CAMPLING, B.G. (1990). Dose response analysis of chemosensitivity testing of small cell lung cancer using the MTT assay. *American Statistical Association, 1989 Proceedings of the Biopharmaceutical Section*, 193–198.

LEE, F.Y.F., SIEMANN, D.W. & SUTHERLAND, R.M. (1989). Changes in cellular glutathione content during adriamycin treatment in human ovarian cancer – a possible indicator of chemosensitivity. *Br. J. Cancer*, **60**, 291–298.

LONG, B.H., MUSIAL, S.T. & BRATTAIN, M.G. (1986). DNA breakage in human lung carcinoma cells and nuclei that are naturally sensitive or resistant to etoposide and teniposide. *Cancer Res.*, **46**, 3809–3816.

LUTZKY, J., ASTOR, M.B., TAUB, R.N., BAKER, M.A., BHALLA, K., GERVASONI, J.E., ROSADO, M., STEWART, V., KRISHNA, S. & HINDENBURG, A.A. (1989). Role of glutathione and dependent enzymes in anthracycline-resistant HL60/AR cells. *Cancer Res.*, **49**, 4120–4125.

MARQUARDT, D. & CENTER, M.S. (1992). Drug transport mechanisms in HL60 cells isolated for resistance to Adriamycin: Evidence for nuclear drug accumulation and redistribution in resistant cells. *Cancer Res.*, **52**, 3157–3165.

MEIJER, C., MULDER, N.H., HOSPERS, G.A.P., UGES, D.R.A. & DEVRIES, E.G.E. (1990). The role of glutathione in resistance to cisplatin in a human small cell lung cancer cell line. *Br. J. Cancer*, **62**, 72–77.

MILROY, R., PLUMB, J.A., BATSTONE, P., MACKLAY, A., WISHART, G.C., HAY, F.G., CANDLISH, W., ADAMSON, R., KHAN, M.Z., BANHAM, S. & KAYE, S.B. (1992). Lack of expression of P-glycoprotein in 7 small cell lung cancer cell lines established both from untreated and from treated patients. *Anticancer Res.*, **12**, 193–200.

MINATO, K., KANZAWA, F., NISHIO, K., NAKAGARA, K., FUJIWARA, Y. & SAIJO, N. (1990). Characterization of an etoposide-resistant human small-cell lung cancer cell line. *Cancer Chemother. Pharmacol.*, **26**, 313–317.

MIRSKI, S.E.L., GERLACH, J.H. & COLE, S.P.C. (1987). Multidrug resistance in a human small cell lung cancer cell line selected in adriamycin. *Cancer Res.*, **47**, 2594–2598.

MISTRY, P., KELLAND, L.R., ABEL, G., SIDHAR, S. & HAPPAP, K.R. (1991). The relationships between glutathione, glutathine-S-transferase and cytotoxicity of platinum drugs and melphalan in eight human ovarian carcinoma cell lines. *Br. J. Cancer*, **64**, 215–220.

MORRISON, D.F. (1976). *Multivariate Statistical Methods*, Second Edition. New York: McGraw-Hill.

MORSTYN, G., RUSSO, A., CARNEY, D.N., KARAWYA, E., WILSON, S.H. & MITCHELL, J.B. (1984). Heterogeneity in the radiation survival curves and biochemical properties of human lung cancer cell lines. *J. Natl Cancer Inst.*, **73**, 801–807.

MOSCOW, J.A., TOWNSEND, A.J. & COWAN, K.H. (1989). Elevation of π class glutathione S-transferase activity in human breast cancer cells by transfection of the GST-π gene and its effect on sensitivity to toxins. *Mol. Pharmacol.*, **36**, 22–28.

NAKAGAWA, K., SAIJO, N., TSUCHIDA, S., SAKAI, M., TSUNOKAWA, Y., YAKOTA, J., MURAMATSU, M., SATO, K., TERADA, M. & TEW, K.D. (1990). Glutathione-S-transferase π as a determinant of drug resistance in transfectant cell lines. *J. Biol. Chem.*, **265**, 4296–4301.

NAKAGAWA, K., YOKOTA, J., WADA, M., SASAKI, Y., FUJIWARA, Y., SAKAI, M., MURAMATSU, M., TERASAKI, T., TSUNOKAWA, Y., TERADA, M. & SAIJO, N. (1988). Levels of glutathione S transferase mRNA in human lung cancer cell lines correlate with the resistance to cisplatin and carboplatin. *Jpn. J. Cancer Res. (Gann)*, **79**, 301–304.

NOONAN, K.E., BECK, C., HOLZMAYER, T.A., CHIN, J.E., WUNDER, J.S., ANDRULIS, I.L., GAZDAR, A.F., WILLMAN, C.L., GRIFFITH, B., VON HOFF, D.D. & RONINSON, I.B. (1990). Quantitative analysis of MDR1 (multidrug resistance) gene expression in human tumors by polymerase chain reaction. *Proc. Natl Acad. Sci. USA*, **87**, 7160–7164.

NYGREN, P., LARSSON, R., GRUBER, A., PETERSON, C. & BERGH, J. (1991). Doxorubicin selected multidrug-resistant small cell lung cancer cell lines characterized by elevated cytoplasmic $Ca^{++}$ and resistance modulation by verapamil in absence of P-glycoprotein overexpression. *Br. J. Cancer*, **64**, 1011–1018.

O'DWYER, P.J., HAMILTON, T.C., YOUNG, R.C., LACRETA, F.P., CARP, N., TEW, K.D., PADAVIC, K., COMIS, R.L. & OZOLS, R.F. (1992). Depletion of glutathione in normal and malignant human cells *in vivo* by buthionine sufoximine – Clinical and biochemical results. *J. Natl Cancer Inst.*, **84**, 264–267.

O'DWYER, P.J., LACRETA, F., NASH, S., TINSLEY, P.W., SCHILDER, R., CLAPPER, M.L., TEW, K.D., PANTING, L., LITWIN, S., COMIS, R.L. & OZOLS, R.F. (1991). Phase I study of thiotepa in combination with the glutathione transferase inhibitor ethacrynic acid. *Cancer Res.*, **51**, 6059–6065.

OZOLS, R.F., LOUIE, K.G., PLOWMAN, J., BEHRENS, B.C., FINE, R.L., DYKES, D. & HAMILTON, T.C. (1987). Enhanced melphalan cytotoxicity in human ovarian cancer *in vitro* and in tumor-bearing nude mice by buthionine sulfoximine depletion of glutathione. *Biochem. Pharmacol.*, **36**, 147–153.

PAGLIA, D.E. & VALENTINE, W.N. (1967). Studies on the quantitative and qualitative characterization of erythrocyte glutathione peroxidase. *J. Lab. Clin. Med.*, **70**, 158–163.

PETERS, W.H.M. & ROELOFS, H.M.J. (1992). Biochemical characterization of resistance to mitoxantrone and Adriamycin in Caco-2 human colon adenocarcinoma cells: A possible role for glutathione S-transferases. *Cancer Res.*, **52**, 1886–1890.

PETERSON, G.L. (1977). A simplification of the protein assay method of Lowry *et al.* which is more generally applicable. *Analyt. Biochem.*, **83**, 346–356.

PUCHALSKY, R. & FAHL, W.E. (1990). Expression of recombinant glutathione-S-transferase-π, Ya, or Yb1 confers resistance to alkylating agents. *Proc. Natl Acad. Sci. USA*, **87**, 2443–2447.

REDDY, C.C., TU, C.-P.D., BURGESS, J.R., HO, C.-Y., SCHOLZ, R.W. & MASSERO, E.J. (1981). Evidence for the occurrence of selenium-independent glutathione peroxidase activity in rat liver microsomes. *Biochem. Biophys. Res. Commun.*, **101**, 970–978.

REEVE, J.G., RABBITTS, P.H. & TWENTYMAN, P.R. (1989). Amplification and expression of mdr1 gene in a multidrug resistant variant of small cell lung cancer cell line NCI-H69. *Br. J. Cancer*, **60**, 339–342.

SABURI, Y., NAKAGAWA, M., ONO, M., SAKAI, M., MURAMATSU, M., KOHNO, K. & KURAWANO, M. (1989). Increased expression of glutathione S-transferase gene in cis-diamminedichloroplatinum(II)-resistant variants of a Chinese hamster ovary cell line. *Cancer Res.*, **49**, 7020–7025.

SAS INSTITUTE INC: SAS/STAT USER'S GUIDE. (1990). Version 6, Fourth Edition. Cary, NC: SAS Institute Inc. pp 209–235.

SCHUURHUIS, G.J., BROXTERMAN, H.J., DE LANGE, J.H.M., PINEDO, H.M., VAN HEIJNINGEN, T.H.M., KUIPER, C.M., SCHEFFER, G.L., SCHEPER, R.J., VAN KALKEN, C.K., BAAK, J.P.A. & LANKELMA, T. (1991). Early multidrug resistance, defined by changes in intracellular doxorubicin distribution, independent of P-glycoprotein. *Br. J. Cancer*, **64**, 857–861.

SEIFTER, E.J. & IHDE, D. (1988). Therapy of small cell lung cancer: A perspective on two decades of clinical research. *Seminars in Oncol.*, **15**, 278–299.

SINGH, S.V., NAU, S., AHMAD, H., AWASTHI, Y.C. & KRISHAN, A. (1989). Glutathione S-transferases and glutathione peroxidases in doxorubicin-resistant murine leukemia P388 cells. *Biochem. Pharmacol.*, **38**, 3505–3510.

SZASZ, G. (1969). A kinetic photometric method for serum gamma glutamyl transpeptidase. *Clin. Chem.*, **15**, 124–136.

TEICHER, B.A., HOLDEN, S.A., KELLEY, M.J., SHEA, T.C., CUCCHI, C.A., ROSOWSKY, A., HENNER, W.D. & FREI, E. III. (1987). Characterization of a human squamous carcinoma cell line resistant to cis-diamminedichloroplatinum (II). *Cancer Res.*, **47**, 388–393.

TIETZE, F. (1969). Enzymatic method for quantitative determination of nanogram amounts of total and oxidized glutathione. *Anal. Biochem.*, **27**, 502–522.

TOWBIN, H., STAEHELIN, T. & GORDON, J. (1979). Electrophoretic transfer of proteins from polyacrylamide gels to nitrocellulose sheets: procedures and some applications. *Proc. Natl Acad. Sci. USA*, **76**, 4350–4354.

WAXMAN, D.J. (1990). Glutathione S-transferases: Role in alkylating agent resistance and possible target for modulation chemotherapy. A review. *Cancer Res.*, **50**, 6449–6454.

WHELAN, R.D.H., HOSKING, L.K., TOWNSEND, A.J., COWAN, K.H. & HILL, B.T. (1989). Differential increases in glutathione S-transferase activities in a range of multidrug-resistant human tumor cell lines. *Cancer Commun.*, **1**, 359–365.

YUSA, K., HAMADA, H. & TSURUO, T. (1988). Comparison of glutathione S-transferase activity between drug-resistant and – sensitive human tumor cells: Is glutathione S-transferase associated with multidrug resistance? *Cancer Chemother. Pharmacol.*, **22**, 17–20.

ZIJLSTRA, J.G. DE VRIES, E.G.E. & MULDER, N.H. (1987). Multifactorial drug resistance in an adriamycin-resistant human small cell lung carcinoma cell line. *Cancer Res.*, **47**, 1780–1784.